# Short Communication: Relationship between Urinary Neutrophil Gelatinase-Associated Lipocalin and Noninfectious Pyuria in Dogs

**DOI:** 10.1155/2015/387825

**Published:** 2015-04-16

**Authors:** D. Proverbio, E. Spada, L. Baggiani, G. Bagnagatti De Giorgi, E. Ferro, P. A. Martino, R. Perego

**Affiliations:** ^1^Dipartimento di Scienze Veterinarie per la Salute, la Produzione Animale e la Sicurezza Alimentare (VESPA), Università degli Studi di Milano, Via Celoria 10, 20133 Milano, Italy; ^2^Dipartimento di Scienze Veterinarie e Sanità Pubblica (DIVET) Università degli Studi di Milano, Via Celoria 10, 20133 Milano, Italy

## Abstract

Neutrophil gelatinase-associated lipocalin (NGAL) is a neutrophil-derived protein whose concentration increases in plasma and urine with ongoing renal damage. Urinary leucocytes can be a potential source of urinary NGAL. The aim of this study is to investigate the effects of urinary neutrophil count and other urinary parameters on urinary NGAL values in urine with negative culture. Urinalysis, urine culture, and determination of urinary NGAL were performed on 33 clinically healthy nonproteinuric dogs with negative urinoculture. The median uNGAL concentration in dogs in this study population was 9.74 ng/mL (IQR 1.93–25.43 ng/mL). In samples with WBCs > 5 hpf (mean 15.9, 6–50 leucocytes/hpf), median uNGAL value was significantly higher than that in samples with WBCs < 5 hpf (mean 0.9, 0–3 leucocytes/hpf), (4.96 pg/mL (0.29–11.34) and 23.65 pg/mL (20.04–29.80), resp.; *P* = 0.0053). The severity of urinary pyuria and the UPC value were correlated with uNGAL concentration. The results of our study show that urinary NGAL concentration is correlated with WBCs number in urinary sediment of dogs with negative urinoculture. The present study suggests that noninfectious pyuria is significantly correlated with urinary NGAL values and might influence uNGAL values.

## 1. Introduction

Neutrophil gelatinase-associated lipocalin (NGAL) is a protein expressed by neutrophils, monocytes, and a variety of tissues such as kidneys, liver, lungs, intestines, and adipose tissue [1, 2]. NGAL is an iron carrier protein that serves as a mediator of the innate immune response in mammals. Bacteria need iron for metabolism. During infection bacterial lipopolysaccharides induce NGAL transcription and bacterial growth is then inhibited by bacteriostatic action of NGAL since it prevents bacterial iron uptake [1, 3].

In physiological condition, renal NGAL is filtered by glomerular and then reabsorbed by the proximal tubular cells. During kidney injury there is a decrease of NGAL reabsorption and an increase of its expression that contributes to an increase of NGAL concentration in both serum and urine [2, 4].

In human patients NGAL has been investigated across a range of several studies [5], assessing that it is induced in proximal tubular epithelial cells during the regenerative process in the kidney after injury [3]. NGAL has been demonstrated to be a highly predictive biomarker for acute kidney injury (AKI), chronic kidney diseases (CKD), and also urinary tract infections (UTI) [5–8].

In veterinary studies urinary NGAL (uNGAL) has been proved to be a valuable biomarker for canine different renal diseases such as AKI and CKD [2, 9–11]. Urine NGAL level seems to be an earlier biomarker than serum creatinine when detecting AKI in dog [12]. A recent study reports that urinary NGAL concentration increases significantly in nonazotemic dogs with urinary tract infection (UTI) [1] and speculates that noninfectious pyuria might influence urinary NGAL values.

The purpose of this study is to evaluate if urinary NGAL concentration is affected by noninfectious pyuria.

## 2. Materials and Methods

### 2.1. Animals

This prospective study was conducted at the Veterinary Teaching Hospital of University of Milan. Blood samples analysis, urinalysis, urine culture, and determination of urinary NGAL were performed on 33 clinically healthy dogs.

Dogs were considered healthy based on the clinical examination and normal results of complete blood count and serum chemistry profile. Urinary tract infection (UTI) was excluded based on the absence of signs of UTI, hematuria, pollakiuria, stranguria or dysuria, negative urine culture, and protein/creatine (UPC) ratio of <0.5. Considering pyuria to be clinically relevant when median WBC in urine sediment/hfp is >5 [13], dogs were divided into 2 group: dogs with mean WBC in urine sediment >5 hfp and dogs with WBC in urine sediment <5 hfp.

### 2.2. Sample Collection

Blood and urine samples were collected as part of routine diagnostic preoperative screening for sterilization surgery and dental scaling. After obtaining informed owner consent surplus urine samples were submitted to uNGAL determination and urine culture.

Urine samples were collected by natural voiding and immediately cooled to 4°C. Routine urinalyses, urine sediment preparations, and quantitative bacteriologic culture of urine were performed in different sections within the Veterinary Teaching Hospital of University of Milan.

### 2.3. Laboratory Methods

Before analysis, urine samples were divided into tree aliquots. Routine urinalysis was performed on all fresh urine samples within 2 hours of submission. Specific gravity of urine samples was measured with a refractometer and urine chemical examination was measured by dipstick tests (Combur10 Test, Roche Diagnostics Indianapolis, IN, USA). One aliquot of each sample was centrifuged at 309 g for 5 minutes and the supernatant was removed, frozen, and stored at −20°C for urine protein, creatinine, and urinary NGAL determination. The remainder of aliquot was used for urine culture within 4 hours of sample collection. Microscopic examination (10 fields at 400x) of centrifuged urine stained sediment was used to identify and quantify the number of leukocytes (WBCs) and red blood cells (RBCs) in the high power field (hpf), crystals, epithelial cells, casts (epithelial cells casts (ECC) and granular casts (GC)), and bacteria.

Protein and creatinine concentrations were measured in the supernatant using a Cobas Mira Classic chemistry analyser (Roche Diagnostic Division, Basel, Switzerland). Urine protein concentration was determined with a Pyrogallol red colorimetric assay (Ben srl Biochemical Enterprise, Milan, Italy) and urine creatinine concentration was determined with the modified kinetic Jaffe method (HAGEN Diagnostica srl, San Giovanni, Italy).

The urine supernatant was diluted in a ratio of  1 : 100 with water before creatinine measurement.

The UPC ratio was calculated automatically by dividing the urine protein concentration (mg/dL) by the creatinine concentration (mg/dL).

Urinary neutrophil gelatinase-associated lipocalin (NGAL) was determined by a commercially available sandwich enzyme-linked immunosorbent assay (ELISA) kit validated for canine species (BioPorto Diagnostics, Denmark). Assays were used in accordance with the manufacturer's instructions. Briefly, after thawing, urine samples aliquots were diluted and either samples or provided standards (NGAL, 0–400 pg/mL) were added in duplicate to 96-well plates and incubated at room temperature for 60 minutes; after incubation and washing, biotinylated Dog-NGAL antibody was dispensed into each microwell and incubated at room temperature for 60 minutes. After washing, the detection antibody HRP-conjugated streptavidin was added and incubated at room temperature for 60 minutes; finally, after washing, colour-forming TMB substrate was added and after 10-minute incubation period the reaction was stopped with sulfuric acid and immediately read. Absorbance was measured at 450 nm. The concentration of NGAL in each sample was calculated from a standard curve, obtained by a 4-parameter regression logistic curve. The detection limit of the ELISA test lies between 4 pg/mL and 400 pg/mL. For statistical calculations values below the detection limit were assigned a value of 0.2 pg/mL.

Intra-assay variability was estimated by determining urinary NGAL concentrations in duplicate on 3 canine urine samples at 3 different concentrations (19.74 pg/mL, 28.52 pg/mL, and 138.39 pg/mL). Duplicate analyses were run twice each day for 5 days. The coefficient of variation, CV, was calculated as mean/DSX100.

### 2.4. Statistical Analysis

Quantitative measurements were described using summary statistics (mean, standard deviation, median, minimum, and maximum).

All data were tested for their normal distribution with D'Agostino-Pearson normality test. Normally distributed data were expressed as mean ± standard deviation. Nonnormally distributed data were expressed as medians with interquartile range (IQR). Pearson's correlation or Spearman's coefficient of rank correlation (rho) were used to evaluate the relationship between uNGAL concentration and urinary parameters: UP/UC, WBCs, RBCs, epithelial cells, crystals, and casts. The statistical difference of uNGAL level between two groups of dogs with WBCs > 5 hpf and with WBCs < 5 hpf and mean between uNGAL concentration in both genders were valuated using independent samples *t*-test or Mann-Whitney independent sample test (MedCalc Software, version 12.7.8.0, Mariakerke, Belgium). Significance was set at *P* < 0.05.

## 3. Results

The dogs group consisted of 33 dogs (22 mixed breeds, 11 mongrels; 20 male, 13 female), with age ranging from 6 months to 14 years (Table 1). The WBCs > 5 hfp group consisted of 12 dogs, 10 intact male, 1 intact female, and 1 neutered female; the WBCs < 5 hfp group consisted of 21 dogs, 3 intact male, 6 neutered male, 4 intact female, and 8 neutered female.

All parameters, except urinary epithelial cells, were not normally distributed.

The median uNGAL concentration in dogs in this study population was 9.74 ng/mL (IQR 1.93–25.43 ng/mL). Median uNGAL value was significantly higher in male than in female (23.37 ng/mL and 4.75 ng/mL, resp.; *P* = 0.0071).

In samples with WBCs > 5 hpf (median 15.9, IQR 6–50 leucocytes/hpf) median uNGAL value was significantly higher than that in samples with WBCs < 5 hpf (median 0.9, IQR 0–3 leucocytes/hpf), 4.96 pg/mL (IQR 0.29–11.34) and 23.65 pg/mL (IQR 20.04–29.80), respectively; *P* = 0.0053. Among considered urinary parameters, only the severity of urinary pyuria and the UPC value were correlated with uNGAL concentration (Table 2). Intra-assay variability was 10.39.

## 4. Discussion

Previous studies have reported that increased uNGAL concentration precedes increase in serum creatinine as marker of acute renal injuries in dogs [10]. NGAL is an emerging renal biomarker for early detection of acute and chronic renal injuries [9–11]. Recently it has been found that uNGAL level increases in presence of UTI [1, 6, 8]. In neutrophils NGAL is present in granules and plays a role in innate immunity to bacterial infection. It has the ability to bind bacterial iron siderophores preventing bacterial growth [1, 6, 8, 11]. UTI is one of the most common bacterial infections in dog [14]. Also in dogs it has been suggested that urinary NGAL can be used as biomarker of UTI [1]. Daure et al. [1] reported that uNGAL concentration significantly increased in nonazotemic dogs with UTI when compared to dogs with negative urine culture results. In Daure et al.'s study, specificity of uNGAL was low (76%) and the authors supposed that the lack of specificity of uNGAL might be due to noninfectious pyuria that might influence uNGAL values. The results of our study show that urinary NGAL concentration is correlated with WBCs number in urinary sediment of dogs with negative urinoculture. This data is further supported by the significant difference found in median uNGAL level between the dog group with WBCs/hpf > 5 and the one with WBCs/hpf < 5. These results can be explained by the presence of NGAL originating from urinary neutrophils.

When uNGAL is used as biomarker to diagnose UTI [1], possible influence of leucocytes should be considered. In most cases of UTI in urinary sediment leucocytes are present [14], but noninfectious pyuria might influence uNGAL values [1]. Sterile pyuria may occur in noninfectious conditions such as urolithiasis or neoplasia [13]. In our study uNGAL was evaluated in urine with negative urine culture, but more than 5 leucocytes/hpf were found in 12 out 33 samples upon examination of urine sediment. We are not able to identify the causes of this noninfectious pyuria. In our study, no correlation between the presence of crystals and leukocytes in the urinary sediment was found. Despite this, the possible presence of stones in the bladder cannot be certainly excluded. A limitation of the current study was that it did not provide further diagnostic investigations such as abdominal ultrasound that could have ruled out the presence of prostatitis in males or neoplasia. We would like to highlight that the dogs group with WBCs > 5 hpf was made almost exclusively of intact males, so it is possible to speculate a prostatic origin of leukocytes. This speculation may also explain the significantly higher median value of uNGAL found in male compared to female (23.37 ng/mL and 4.75 ng/mL resp.; *P* = 0.0071).

Furthermore one of the limitations of this study is the use of urine obtained by spontaneous urination that may have interfered with the presence of leukocytes, as the WBCs, could result from contact with the genital mucosa or skin.

As already described in human and veterinary medicine [9, 15] in this study we found a positive correlation between UPC value and uNGAL concentration, although all the subjects considered in this study were nonproteinuric.

## 5. Conclusion

Although limited by the reduced number of cases, our results suggest that, as urinary neutrophils can be regarded as a potential source of uNGAL, also noninfectious pyuria could contribute to urinary NGAL concentration. Therefore the presence of noninfectious pyuria may affect interpretation of uNGAL results, causing misdiagnosis of UTI if uNGAL was used as marker disease. This result must be confirmed on a larger number of samples that should be collected in subjects in which disorders that may cause sterile pyuria were diagnosed.

## Figures and Tables

**Table 1:** Age, sex, breed, surgery, and urinary NGAL concentration in 33 dogs of the study.

Age	Sex	Breed	Surgery	uNGAL (pg/mL)
6	M	Poodle	Dental scaling	3.43
9	FC	Pitt bull	Dental scaling	6.54
4	F	G. shepherd	Neutering	18.23
14	MC	Mongrel	Dental scaling	2.68
2.5	M	Poodle	Neutering	21.64
6	MC	Labrador	Dental scaling	25.43
0.7	M	Rottweiler	Neutering	19.51
3	M	Rottweiler	Neutering	30.58
11	FC	Mongrel	Dental scaling	0.2
3	M	Dachshund	Neutering	75.61
4	MC	Mongrel	Dental scaling	0.2
5	F	Mongrel	Neutering	27.48
3	F	Mongrel	Neutering	27.25
12	MC	Mongrel	Dental scaling	33.86
13	F	Sp. spaniel	Dental scaling	9.74
11.3	FC	Mongrel	Dental scaling	9.1
6	MC	Greyhound	Dental scaling	6.13
3	M	Greyhound	Neutering	24.86
4	M	Mongrel	Neutering	25.44
0.6	F	J. Russell	Neutering	1.54
3	FC	Mongrel	Dental scaling	4.75
3	M	Mongrel	Neutering	23.1
5	M	Sp. spaniel	Neutering	0.29
6	FC	Labrador	Dental scaling	0.2
12	FC	Golden R.	Dental scaling	5.18
0.6	M	Mongrel	Neutering	23.65
7	FC	Mongrel	Dental scaling	0.2
6	FC	Cocker sp.	Dental scaling	0.2
4	FC	Labrador	Dental scaling	2.07
6	M	Poodle	Neutering	1.22
7.6	M	Boxer	Neutering	32.28
11	MC	Setter	Dental scaling	57.96
8	M	Dachshund	Neutering	11.34

**Table 2:** Correlation between urinary variables and uNGAL concentration on 33 healthy dogs.

Variable	Sample	Median (IQR)	Spearman's coefficient (rho)^**^
	UPC	0.04 (0.02–0.16)	rho = 0.572 *P * = 0.0014
	WBCs	2 (0–10)	rho = 0.561 *P* = 0.0015
uNGAL	Epithelial cells^*^	0.75 ± 1^*^	rho = 0.205 *P* = 0.2529
	Crystals	0 (0-1)	rho = 0.101 *P* = 0.5752
	Casts	0 (0–2)	rho = 0.187 *P* = 0.2964

^*^Mean ± standard deviation.

^**^rho: nonparametric measure of the strength and direction of association between two variables.

IQR: interquartile range.

*P* < 0.005 is considered significant.
